# Monitoring and assessment of spatiotemporal soil salinization in the Lake Urmia region

**DOI:** 10.1007/s10661-024-13055-6

**Published:** 2024-09-20

**Authors:** S. Mirzaee, A. Mirzakhani Nafchi, Y. Ostovari, M. Seifi, S. Ghorbani-Dashtaki, H. Khodaverdiloo, S. Chakherlou, R. Taghizadeh-Mehrjardi, B. Raei

**Affiliations:** 1 Department of Agronomy, Horticulture and Plant Sciences, College of Agriculture, Food and Environmental Sciences, South Dakota State University, Brookings, SD 57007 USA; 2 Departments of Agricultural & Biosystem Engineering, College of Agriculture, Food & Environmental Sciences, South Dakota State University, Brookings, SD 57007 USA; 3 Department of Soil Science, Faculty of Agriculture, Shiraz University, Shiraz, Iran; 4 Department of Soil Science, Faculty of Agriculture, Tabriz University, Tabriz, Iran; 5 Department of Soil Science, College of Agriculture, Shahrekord University, Shahrekord, Iran; 6 Department of Soil Science, Faculty of Agriculture, Urmia University, Urmia, Iran; 7 School of Agriculture and Food Sciences, The University of Queensland, Brisbane, QLD Australia; 8 Faculty of Agriculture and Natural Resources, Ardakan University, Ardakan, Iran

**Keywords:** Modelling, Remote sensing, Spectral indices, Soil salinity

## Abstract

Soil salinization stands as a prominent global environmental challenge, necessitating enhanced assessment methodologies. This study is dedicated to refining soil salinity assessment in the Lake Urmia region of Iran, utilizing multi-year data spanning from 2015 to 2018. To achieve this objective, soil salinity was measured at 915 sampling points during the 2015–2018 timeframe. Simultaneously, remote sensing data were derived from surface reflectance data over the same study period. Four distinct scenarios were considered such as a newly developed spectral index (Scenario I), the newly developed index combined with other salt-based spectral indices from the literature (Scenario II), indirect spectral indices based on vegetation and soil characteristics (Scenario III), and the amalgamation of both direct and indirect spectral indices (Scenario IV). Linear Regression (LR), Support Vector Machine (SVM), and Random Forest (RF) were employed to assess soil salinity. The measured data divided to 75% of the data as the calibration dataset, while the remaining 25% constituted the validation dataset. The findings revealed a correlation between soil salinity and spectral indices from the literature, with a range of -0.53 to 0.51, while the newly developed spectral index exhibited a stronger correlation (r = 0.59). Furthermore, RF yielded superior results when using the newly developed spectral index (Scenario I). Overall, SVM emerged as the most effective model (ME = -9.678, R^2^ = 0.751, and RPIQ = 1.78) when integrating direct and indirect spectral indices (Scenario IV). This study demonstrates the efficacy of combining machine learning techniques with a blend of newly developed and existing spectral indices from the literature for the monitoring of soil salinity, particularly in arid and semi-arid regions.

## Introduction

Geology, its associated chemistry, local hydrology, and climate represent pivotal factors contributing to soil salinization (Hopman et al., 2021). Generally, soil salinization occurs in regions where evapotranspiration significantly surpasses precipitation, resulting in the accumulation of salts within the soil matrix, which subsequently migrate both vertically and horizontally in response to water movement (Wang et al., 2022). The shifting climate patterns have propelled soil salinization to become one of the most prevalent environmental challenges, particularly in arid and semi-arid areas. These concerns extend across various global regions, including the USA (Lal, 2004; Eldeiry & Garcia, 2008; Scudiero et al., 2014), China (Ren et al., 2019; Wang et al., 2019, 2020), Turkey (Bahceci & Nacar, 2007; Gorji et al., 2017; Kaya et al., 2022), Iran (Gorji et al., 2020; Seifi et al., 2020; Taghadosi et al., 2019; Taghizadeh-Mehrjardi et al., 2021), Saudi Arabia (Tripathi et al., 1997; Allbed et al., 2014), in Sudan (Sulieman et al., 2023) and India (Singh, 2014). Of particular note is the Lake Urmia region in Iran, housing a hypersaline lake that poses a significant threat to land resources and ecosystem health (Gorji et al., 2020; Seifi et al., 2020; Taghizadeh-Mehrjardi et al., 2021). Soil resources in the Lake Urmia region, especially within the context of saline seeps, salt dust, and human-induced factors, are undergoing degradation. The distribution of salt content in field conditions exhibits neither temporal constancy nor uniformity in soil depth. As such, precise monitoring, prediction, and assessment of the spatio-temporal dynamics of soil salinization in the Lake Urmia region are paramount for advancing land resource management and safeguarding ecosystem health.

Traditionally, soil salinity is ascertained by measuring electrical conductivity in the laboratory, involving the extraction of saturated soil solutions (Richards, 1954). However, this direct measurement approach is cumbersome, costly, and time-consuming when applied at a large scale (Seifi et al., 2020; Wang et al., 2022). In contrast, remote sensing emerges as a practical and efficient method, offering real-time insights into changes in soil salinities (Fern´andez-Buces et al., 2006; Kaya et al., 2022; Sulieman et al., 2023; Wang et al., 2022). Remote sensing data allows for rapid monitoring and mapping of soil salinity across diverse spatial and temporal scales. Utilizing remote sensing information, saline soils can be detected based on distinctive reflectance patterns (Lopes et al., 2020). Soil salinity can be directly determined by leveraging original bands and their combinations, especially in the visible to near-infrared (NIR) spectral range. In this context, the reflectance levels increase with higher salt content, providing a foundational basis for the identification and monitoring of soil salinity through reflectance data (Metternicht & Zinck, 2003).

While original satellite bands have offered some success in soil salinity determination, they often fall short of providing precise results (Wang et al., 2018). To enhance predictive accuracy, various spectral indices have been developed for digital soil mapping (DSM) of soil parameters (Fathizad et al., 2020). Early investigations, such as that of Tripathi et al. (1997), demonstrated the improved efficacy of spectral indices generated by combining visible light and NIR bands to identify salt content in bare soils. Subsequent research endeavors led to the creation of various spectral indices aimed at determining salt content, including Salinity Index 1–3 (Khan et al., 2005) and Soil Salinity and Sodicity Indices 1–2 (Bannari et al., 2008). Furthermore, Douaoui et al. (2006) and Chen et al. (2021) highlighted the direct impact of salt on vegetation health in areas with vegetation cover. As a result, soil salinity can be indirectly predicted by assessing vegetation growth status. In alignment with this perspective, studies by Seifi et al. (2020) and Nabiollahi et al. (2021) indicated that the original bands, particularly the green, red, and NIR bands, can be utilized to determine vegetation type, growth status, and, indirectly, salt content. Building upon this foundation, several spectral indices have been formulated to detect soil salinity, including the Canopy Response Salinity Index (Scudiero et al., 2014), Salinity Index IV-X (Abbas & Khan, 2007), and Salinity Ratio Index (Metternicht & Zinck, 2003).

Recent research efforts have sought to correlate spectral indices with statistical and machine learning methods. Studies by Davis et al. (2019), Seifi et al. (2020), and Wang et al. (2021) applied regression models to estimate soil salinity using spectral indices. These endeavors revealed that multiple linear regression models provide an intuitive representation of the relationship between the dependent variable (i.e., soil salinity) and independent variables (i.e., spectral indices), offering strong interpretability. Furthermore, some researchers have employed machine learning-based models for soil salinity estimation (Cao et al., 2022; Hoa et al., 2019; Keshavarzi et al., 2023; Sulieman et al., 2023; Taghizadeh-Mehrjardi et al., 2021). It has been demonstrated that machine learning models present distinct advantages for modeling soil salinity, particularly when a significant number of covariates are involved (Keshavarzi et al., 2023; Sulieman et al., 2023; Taghizadeh-Mehrjardi et al., 2021). However, one of the primary limitations of machine learning models is their propensity to operate as "black box" models, often lacking the transparency needed to interpret the contribution of individual covariates (Wang et al., 2021).

The Lake Urmia region hosts diverse situations, ranging from bare soil to unsowed soil and cultivated soil managed with various agricultural practices. These variations necessitate the development of a new spectral index integrated with a precise machine learning method to predict soil salinity accurately in this region. Additionally, the presence of different crops and natural vegetation, each with varying salt tolerance levels and subject to a spectrum of abiotic and biotic stresses, can influence the reflectance patterns of vegetation. Consequently, the utilization of multi-year reflectance data becomes crucial for deriving a robust predictive model. The objectives of present study were to: (i) evaluate both developed direct spectral indices (salt-based indices) and indirect spectral indices from existing literature, (ii) formulate a novel spectral index that enhances the precise estimation of salt content by leveraging multi-year reflectance data, and (iii) Predict salt content through the integration of spectral indices, regression, and machine learning methodsand map soil salinity in the Lake Urmia region using the most effective methodology available.

## Material and methods

### Study area

**Fig. 1:**
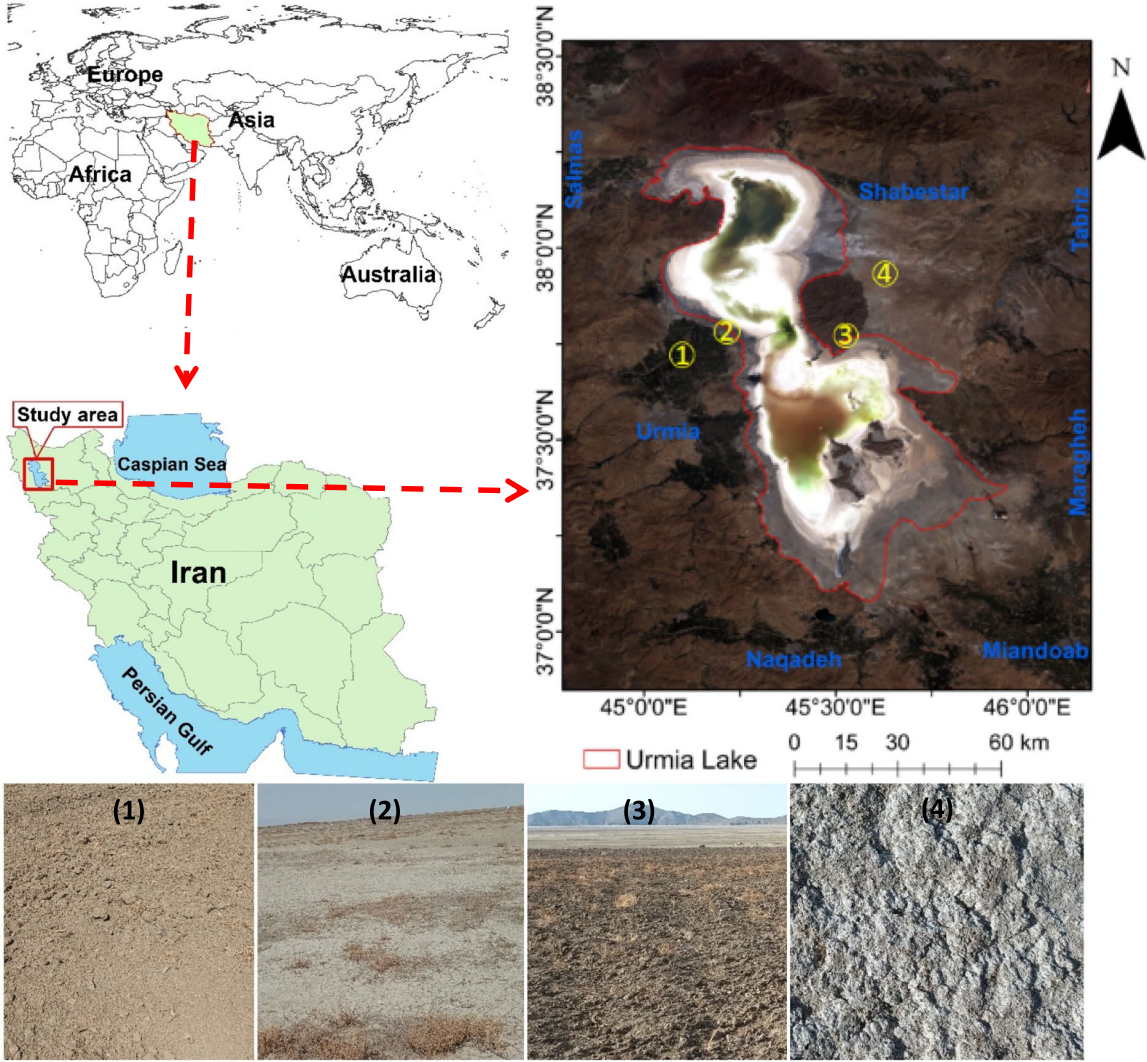
Location of study area in northwestern Iran

The study was conducted in the Lake Urmia region, situated between latitudes 36° 52′ and 38° 31′ N and between 44° 47′ and 46° 12′ E (Fig. 1). Lake Urmia, also known as Urmu gölü in Turkish, is located in northwestern Iran and represents one of the saltiest lakes globally (Eishoeei et al., 2019). The lake's dimensions vary, with a length ranging from 130 to 146 km and a width spanning from 15 to 58 km. The narrowest section, measuring 15 km, is situated between Islami Island and Zanbil Mountain, connected by a constructed causeway. Being a closed basin lake, Lake Urmia experiences fluctuations in water levels in response to variations in precipitation and evaporation. Several rivers contribute to the main water source of Lake Urmia, including Simineh, Zarrineh, Aji Chay, Barandouz, and Nazlou rivers (Sharifi et al., 2018). Notably, the lake's northern region boasts greater depth compared to the southern region, evident in recent satellite imagery depicting the initial recession of water from the southern section. A sustained reduction in water inflow has led to a gradual decrease in the lake's surface area since 1995, with Mardi et al. (2018) reporting a substantial 90% reduction in the lake's area over recent decades. Various factors have contributed to the desiccation of the lake, including extensive dam construction, intensified agricultural activities, urban expansion, recurring droughts resulting from severe climate change, and the construction of a causeway that has altered the lake's hydrological system (Alborzi et al., 2018). Consequently, the salinity levels of the lake have markedly increased in recent years, impacting ecosystem health, local agriculture, and tourism in the region.

### Meteorology

The study area in Iran features a semi-arid climate characterized by mild springs, hot and arid summers, cool autumns, and cold winters. The Lake Urmia region experiences an average annual temperature of 13.5 ^◦^C, with mean annual precipitation measuring 450 mm (Panahi et al., 2020). Precipitation primarily occurs in late autumn, winter, with snowfall, and especially during the spring season.

### Topography

**Fig. 2:**
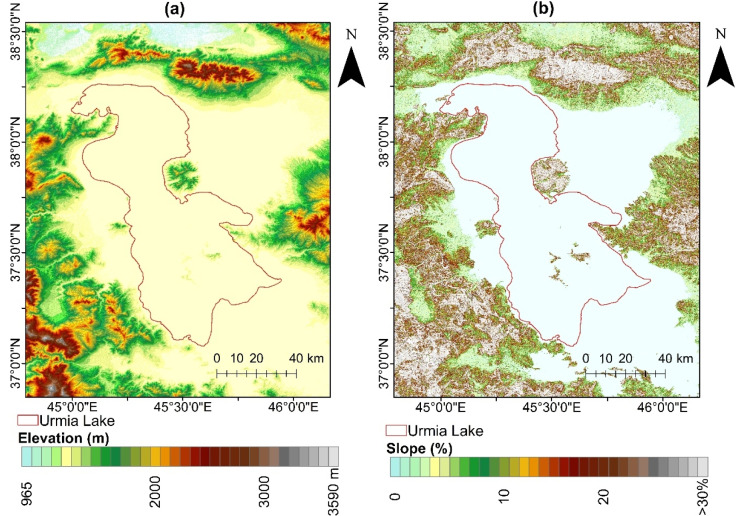
The maps of morphometric indices including elevation (**a**) and slope (**b**)

The topographical characteristics of the study area, as determined from a digital elevation model (DEM with a 30-m resolution), encompass factors such as elevation and slope, illustrated in Fig. 2. As depicted in topographic maps (Fig. 2), the Lake Urmia region primarily consists of piedmont plains featuring gentle slopes. In the proximity of the lake, the terrain gradually transitions to steeper inclines leading to the adjacent mountains. It is noteworthy that topographical factors significantly influence the occurrence of saline seeps and salt dust in the vicinity of the lake.

### Remote sensing data

#### Landsat data

The Landsat 8 satellite is the product of a collaborative effort between NASA (National Aeronautics and Space Administration) and the USGS (U.S. Geological Survey). Launched on February 11, 2013, from Vandenburg Air Force Base, California, this satellite operates in a sun-synchronous, near-polar orbit at an inclination of 98.2 degrees, maintaining an altitude of 705 km (Roy et al., 2014). Landsat 8 is equipped with two primary sensors: the OLI (Operational Land Imager) and the TIRS (Thermal Infrared Sensor)(Roy et al., 2014). The satellite offers repeated coverage every 16 days, with each scene covering an area of 185 km × 180 km.

For the current study, cloud-free Landsat 8 level-1 images captured in the month of August from the years 2013 to 2022 were obtained from the U.S. Geological Survey (https://www.usgs.gov/). The study area spans three scenes of Landsat images, identified by the path/row combinations 168/34, 169/33, and 169/34.

#### Image pre-processing

In the present study, the corrections were geometric and radiometric corrections. In order to correct geometric distortion, all of satellite images were georeferenced using control points obtained from GPS. Moreover, radiometric corrections are necessary to mitigate image noise stemming from sensor, processing, and atmospheric effects. First, the $\text{DN}(\lambda )$ (digital number) of raw images is converted to radiance based on following equation (Chander et al., 2009; El Harti et al., 2016):

1 $$L\left(\lambda \right)=Grain\left(\lambda \right)+DN\left(\lambda \right)+Offset(\lambda )$$

where $\lambda$ is wavelength (µm), $L\left(\lambda \right)$ is radiance at the sensor’s aperture [W/(m^2^ µm sr], $Grain\left(\lambda \right)$ is band-specific rescaling gain factor [(W/m^2^ sr µm)/DN], and $Offset(\lambda )$ is band-specific rescaling offset or bias factor [W/(m^2^ µm sr]. In the second stage, the radiance is converted to surface reflectance through atmospheric correction. After that, the $\rho \left(\lambda \right)$ (reflectance at the surface) was calculated (Chander et al., 2009; El Harti et al., 2016).

The radiance and atmospheric noise were removed using *RStoolbox* (Leutner & Horning, 2023) and *raster* (Hijmans, 2023) packages in R software. Finally, the three images with path/row combinations 168/34, 169/33 and 169/34 for each year were mosaiced.

#### Spectral indices in the literature

Numerous spectral indices, including those reliant on salt, vegetation, and soil-related parameters, have been developed and documented in the existing scientific literature. A comprehensive listing of these indices is provided in Table 1.

**Table 1:** Landsat OLI bands and developed spectral indices in the literature

Catalog	Explanation	Abbreviation	Formula	Reference
Vegetation indices	Normalized difference vegetation index	NDVI	(${\rho }_{NIR}-{\rho }_{R})/({\rho }_{NIR}+{\rho }_{R})$	Rouse et al. (1974)
	Soil adjusted vegetation index	SAVI	$2\left({\rho }_{NIR}-{\rho }_{R}\right)/({\rho }_{NIR}+{\rho }_{R}+1)$	Huete (1988)
	Vegetation soil salinity index	VSSI	$2{\rho }_{G}-5({\rho }_{R}+{\rho }_{NIR})$	Dehni and Lounis (2012)
	Enhanced vegetation index	EVI	$2.5({\rho }_{NIR}-{\rho }_{R})/({\rho }_{NIR}+6{\rho }_{R}+7.5{\rho }_{B}+1)$	Huete (1988)
	Non-linear vegetation index	NLVI	$({{\rho }_{NIR}}^{2}-{\rho }_{R})/({{\rho }_{NIR}}^{2}+{\rho }_{R})$	Goel and Qin (1994)
	Differential vegetation index	DVI	${\rho }_{NIR}-{\rho }_{R}$	Tucker (1979)
	Green ratio vegetation index	GRVI	${\rho }_{\text{NIR}}/{\rho }_{G}$	Sripada et al. (2006)
Salinity indices	Salinity index	SI-T	$({\rho }_{R}/{\rho }_{NIR})100$	Tripathi et al. (1997)
	Salinity index	SI	${({\rho }_{B}\times {\rho }_{R})}^{0.5}$	Douaoui et al. (2006)
	Salinity index 1	SI1	${({\rho }_{G}\times {\rho }_{R})}^{0.5}$	Khan et al. (2001)
	Salinity index 2	SI2	${({{\rho }_{G}}^{2}+{{\rho }_{R}}^{2}+{{\rho }_{NIR}}^{2})}^{0.5}$	Khan et al. (2005)
	Salinity index 3	SI3	${({{\rho }_{G}}^{2}+{{\rho }_{NIR}}^{2})}^{0.5}$	Manière et al. (1993)
	Salinity index 4	SI4	${\rho }_{SWIR1}/{\rho }_{NIR}$	Douaoui et al. (2006)
	Salinity index I	S1	${\rho }_{B}/{\rho }_{R}$	Abbas and Khan (2007)
	Salinity index II	S2	$({\rho }_{B}-{\rho }_{R})/({\rho }_{B}+{\rho }_{R})$	Abbas and Khan (2007)
	Salinity index III	S3	${\rho }_{G}\times {\rho }_{R}/{\rho }_{B}$	Abbas and Khan (2007)
	Salinity index V	S5	${\rho }_{B}\times {\rho }_{R}/{\rho }_{G}$	Abbas and Khan (2007)
	Salinity index VI	S6	${\rho }_{R}\times {\rho }_{NIR}/{\rho }_{G}$	Abbas and Khan (2007)
	Enhanced residues soil salinity index	ERSSI	${{\rho }_{G}}^{2}/{\rho }_{B}\times {\rho }_{SWIR1}$	Wang et al. (2022)
	Canopy response salinity index	CRSI	${(({\rho }_{NIR}\times {\rho }_{R}-{\rho }_{G}\times {\rho }_{B})/({\rho }_{NIR}\times {\rho }_{R}+{\rho }_{G}\times {\rho }_{B}))}^{0.5}$	Scudiero et al. (2014)
Soil indices	Clay index	CI	${\rho }_{SWIR1}/{\rho }_{SWIR2}$	Drury (1987)
	Gypsum index	GI	$({\rho }_{SWIR1}-{\rho }_{SWIR2})/({\rho }_{SWIR1}+{\rho }_{SWIR2})$	Nield et al. (2007)
	Brightness index	BI	${({{\rho }_{G}}^{2}+{{\rho }_{B}}^{2})}^{0.5}$	Tripathi et al. (1997)
	Normalized multi-band drought index	NMDI	${\rho }_{NIR}-\left({\rho }_{SWIR1}-{\rho }_{SWIR2}\right)/{\rho }_{NIR}+\left({\rho }_{SWIR1}-{\rho }_{SWIR2}\right)$	Wang and Qu (2007)

### Soil sampling and analysis

**Fig. 3:**
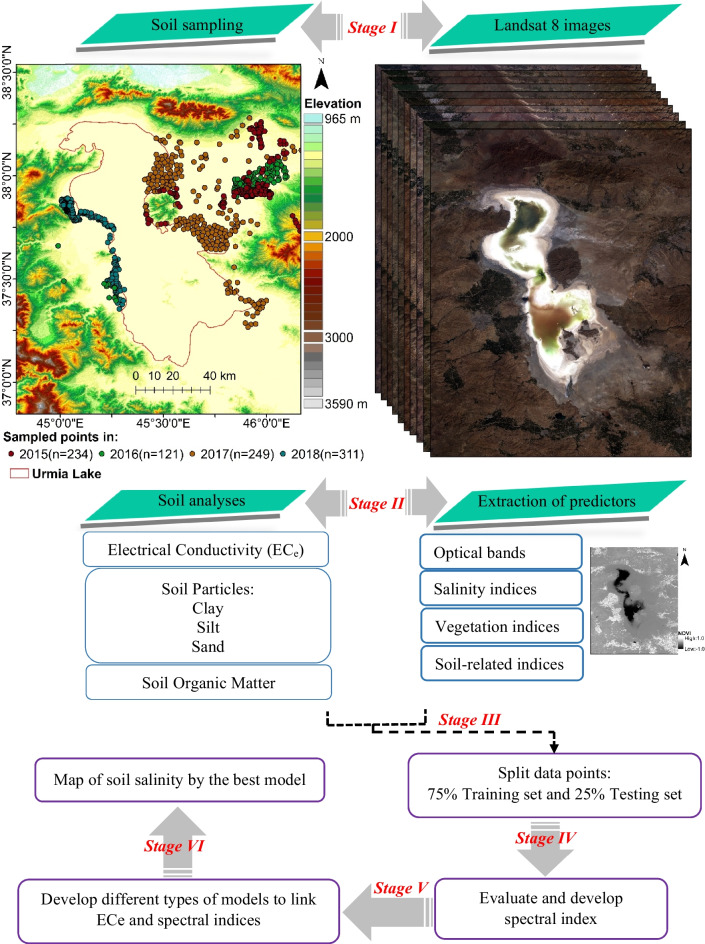
Detailed flowchart of methodology for predicting soil salinity in the Lake Urmia region

Sampling activities in this region were conducted during the months of July and August from 2015 to 2018. A total of 915 sampling points were systematically chosen, with the distribution being 234 points in 2015, 121 points in 2016, 249 points in 2017, and 311 points in 2018. The selection of these points took into account factors such as soil types, land use patterns, and insights gained from previous field sampling experiences, as illustrated in Fig. 3. The most common soil types are Inceptisols, Entisols, and Mollisols in this area according to the Soil Taxonomy (USDA, 2010).

At each sampling point, a bulk sampling strategy was employed. This method involved the collection of three sub-soil samples, each extracted at a spatial resolution equivalent to the 30-m scale of Landsat Visible-Near Infrared (VIS–NIR) bands, and specifically taken from the topsoil layer (0–20 cm). Subsequently, these sub-samples were homogenized to form a composite sample. All collected soil samples underwent a comprehensive laboratory process, which included thorough air-drying. During this drying process, any remnants of vegetation and extraneous materials such as stones were diligently removed. Following this preparation, the electrical conductivity (ECe) was assessed to determine soil salinity. This measurement was performed through the extraction of saturated soil at an ambient room temperature of 25 °C, in accordance with established procedures (U.S. Salinity Laboratory Staff, 1954). Furthermore, the soil organic carbon (SOC) content was quantified utilizing a wet-oxidation method as outlined by Nelson (1982). The soil composition in terms of clay (< 0.002 mm), silt (0.002–0.50 mm), and sand (0.50–2.0 mm) was determined using a hydrometer-based methodology, as described by Gee and Bauder (1986). A visual representation of the sampling process is presented in Fig. 3.

### Modelling soil salinity by regression and machine learning methods

#### Linear regression

Linear regression is a statistical technique employed for the prediction of unknown data (Mirzaee et al., 2016), such as soil salinity in this context. It hinges on utilizing independent variables, which in this study comprise remotely sensed data, as predictors. The linear regression model is delineated as follows (Ho, 2006):

2 $$Y\left(i\right)={\beta }_{0}+\sum_{k}{\beta }_{k}{X}_{ik}+{\varepsilon }_{i}$$

where $Y\left(i\right)$ is the dependent variable (in present study: soil salinity), ${X}_{ik}$ are the independent variables (in present study: spectral indices) at the *i* location and *k* is the number of independent variables, ${\beta }_{0}$ is the intercept, ${\beta }_{k}$ is the regression coefficients, and ${\varepsilon }_{i}$ is error at *i* location. The objective of developing a linear regression is to optimize regression coefficients (i.e. *β*) by applying the following equation.

3 $$\underset{\beta }{\text{min}}{\Vert Y-X\beta \Vert }_{2}^{2}$$

where subscript L2 indicates the L2-norm of the vector. To drive a linear regression model, it is assumed that the distribution of errors is normal. Additionally, it is assumed that the average of errors is zero ($E\left(\varepsilon \right)=0$) and variance of errors is the same ($Var\left(\varepsilon \right)={\sigma }^{2}$). Furthermore, it is assumed that the error data are independent, i.e., ${\varepsilon }_{i}$ has no effects on the others error data (${\varepsilon }_{i+1}$). Moreover, a VIF test (variance inflation factors) was employed for checking multi-collinearity in developed regression models by *mctest* package in R software. The input variables, with VIF > 10, were considered as highly correlated variables and removed (Curto & Pinto, 2011). The regression model was developed by using *Stats* package in R software.

#### Support vector machine

The SVM is one of the machine learning models, that learn according to statistical theory. In this method to project data into a high-dimensional feature space, kernel functions were used (Forkuor et al., 2017). In this study, the Radial Basis Function (RBF) was employed duo to accurate results in agricultural research (Gasmi et al., 2022; Keskin et al., 2019) following as:

4 $$k\left({x}_{i},{x}_{j}\right)=\text{exp}(-\sigma {\Vert {x}_{i}+{x}_{j}\Vert }^{2})$$

where x represents input variables, σ shows width of the Radial Basis Function, and k represents a user-defined kernel function (Jeong et al., 2017). The key hyperparameters of this method are kernel (RBF in this study), regularization parameter (C), kernel coefficient (σ), and a margin of tolerance (ɛ). In this machine learning model, optimizing σ parameter is of crucial importance. The SVM model was developed using *kernlab* package in R software.

#### Random forest

RF (Random Forest) is a machine learning algorithm developed by Breiman (2001). It can aggregate ideas and solve the problems in the regression and classification methods. In this method, the importance of variables is determined by two important parameters including (i) the number of trees in the forest and (ii) the size of the input variables subset. RF performance is primarily compared using the out-of-bag (OOB) error (Genuer et al., 2010). The variable importance (*VI*) of X^i^ in this method is calculated as follows:

5 $$VI\left({X}^{i}\right)=\frac{1}{ntree}{\sum }_{t}(\varepsilon {\widehat{OOB}}_{t}^{i}-\varepsilon {OOB}_{t})$$

Where *ntree* indicates the number of trees in the forest, $\varepsilon {\widehat{OOB}}_{t}^{i}$ represents the error at an especial tree (i.e. *t*) and $\varepsilon {OOB}_{t}$ shows a perturbed sample affected by the permuted values of X^i^ (Wang et al., 2019). In this method, the hyperparameters are number of trees, maximum depth, minimum samples split, minimum samples leaf, and maximum features. The RF model was developed using *randomForest* package in R software.

### Input variables for developing different types of methods.

The input variables in the present study were (Table 2):

(i) Scenario I: Newly developed spectral index in present study,
(ii) Scenario II: Direct spectral indices (i.e. salt-based indices),
(iii) Scenario III: Indirect spectral indices such as vegetation- and soil-based indices, and
(iv) Scenario IV: Direct plus indirect spectral indices.

**Table 2:** The arrangement of input variables in different scenarios to estimate soil salinity

Scenario	New index	Direct indices	Indirect indices
Scenario I	+	-	-
Scenario II	-	+	-
Scenario III	-	-	+
Scenario IV	-	+	+

+ Shown inputs variables to estimate soil salinity, while—means not.

### Statistical analysis

In order to create models that possess both generality and robustness, the collected sampling points were subjected to a random division process (Gasmi et al., 2021). This division entailed allocating 75% of the data as the calibration dataset, while the remaining 25% constituted the validation dataset. This partitioning procedure was conducted through the utilization of the *caTools* package in the R software environment, as visually represented in Fig. 3. In the present study, three common statistic criteria such as (i) ME (mean error), (ii) R^2^ (coefficient of determination), and (iii) RPIQ (ratios of performance to inter-quartile distance) were employed to compare and evaluate the performance of the developed model (Cao et al., 2022).

## Results and discussion

### The characteristics of soil salinity in Lake Urmia region

Table 3 presents the basic statistics of measured soil properties, including soil organic carbon, clay, silt, and sand, in the Lake Urmia region. Additionally, descriptive statistics for electrical conductivity (ECe) measurements conducted from 2015 to 2018 in various parts of the study area are provided in Table 3. As observed in Table 3, the measured ECe values for the entire dataset ranged from 0.13 to 557.0 dS m^−1^ in the Lake Urmia region, with an average value of 65.79 dS m^−1^. The data obtained in this region exhibited a high degree of variation, as indicated by a coefficient of variation (CV) of 166.18% (Table 3). Table 4 presents the ECe values of sampled points categorized based on the U.S. Salinity Laboratory classification (U.S. Salinity Laboratory Staff, 1954). In Table 4, it is shown that 27.4% of the sampled points were categorized as non-saline soils (0 to 2 dS m^−1^), 9.3% as slightly saline soils (2 to 4 dS m^−1^), 10.7% as moderately saline soils (4 to 8 dS m^−1^), 8.2% as strongly saline soils (8 to 16 dS m^−1^), and 44.4% as extremely saline soils (> 16 dS m^−1^). Consequently, the substantial variation in the measured ECe data, coupled with multi-year reflectance data spanning from 2015 to 2018, enhances the generalizability of the developed models.

**Table 3:** Descriptive statistics of measured soil properties

Soil properties		Minimum	Maximum	Mean	Standard deviation	CV (%)
Clay (%)		5.0	56.2	23.3	11.16	47.94
Silt (%)		8.7	75.6	42.45	12.28	28.92
Sand (%)		6.2	76.2	37.73	18.32	48.57
SOC (%)		0.20	3.71	1.99	0.95	47.60
EC_e_ (dS m^−1^) in:						
	2015	0.60	130.90	19.03	26.36	138.46
	2016	0.32	155.00	22.19	36.48	164.41
	2017	0.62	557.00	184.66	149.69	80.97
	2018	0.13	162.00	24.53	34.59	141.02
	Total	0.13	557.00	65.79	109.3	166.18

SOC is soil organic carbon and EC_e_ is electrical conductivity

**Table 4:** Distribution of sampled points (n = 915) in different soil salinity classes

Soil salinity	2015	2016	2017	2018	Total
Non-saline (0 to 2 dS M^−1^)	54	25	29	141	249
Slightly saline (2 to 4 dS M^−1^)	29	25	12	19	85
Moderately saline (4 to 8 dS M^−1^)	38	25	13	21	97
Strongly saline (8 to 16 dS M^−1^)	44	15	10	11	80
Extremely saline (> 16 dS M^−1^)	69	31	185	119	404

### Spectra analysis

**Fig. 4:**
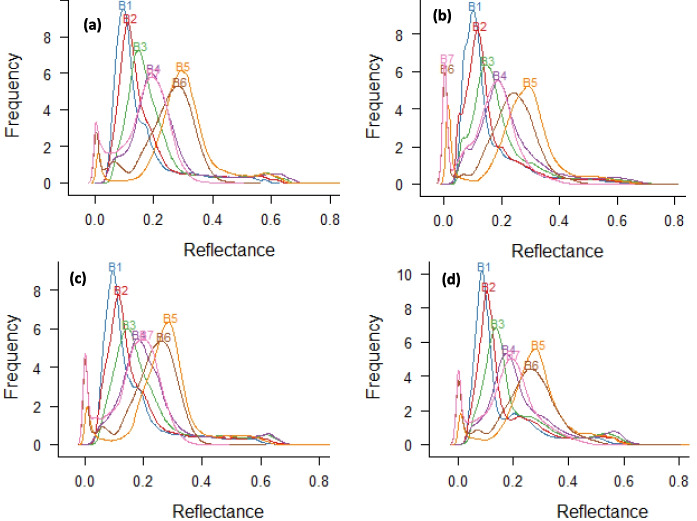
Density distribution of the reflectance in different spectral bands (B1, B2, B3, B4, B5, B6, and B7 indicate the ${\rho }_{CA}$, ${\rho }_{B}$, ${\rho }_{G}$, ${\rho }_{R}$, ${\rho }_{NIR}$, ${\rho }_{SWIR1}$, and ${\rho }_{SWIR2}$, respectively) for 2015 (**a**), 2016 (**b**), 2017 (**c**), and 2018 (**d**)

The distribution of reflectance values in various spectral bands of satellite images for the years 2015, 2016, 2017, and 2018 is illustrated in Fig. 4. The reflectance values across spectral bands in different years ranged from 0 to 0.8 (Fig. 4). Each subfigure in Fig. 4 demonstrates variations in minimum, maximum, and mean reflectance values for their respective spectral bands. These differences can be attributed to various environmental factors, including climatic conditions, agricultural practices, and other variables specific to each year.

**Fig. 5:**
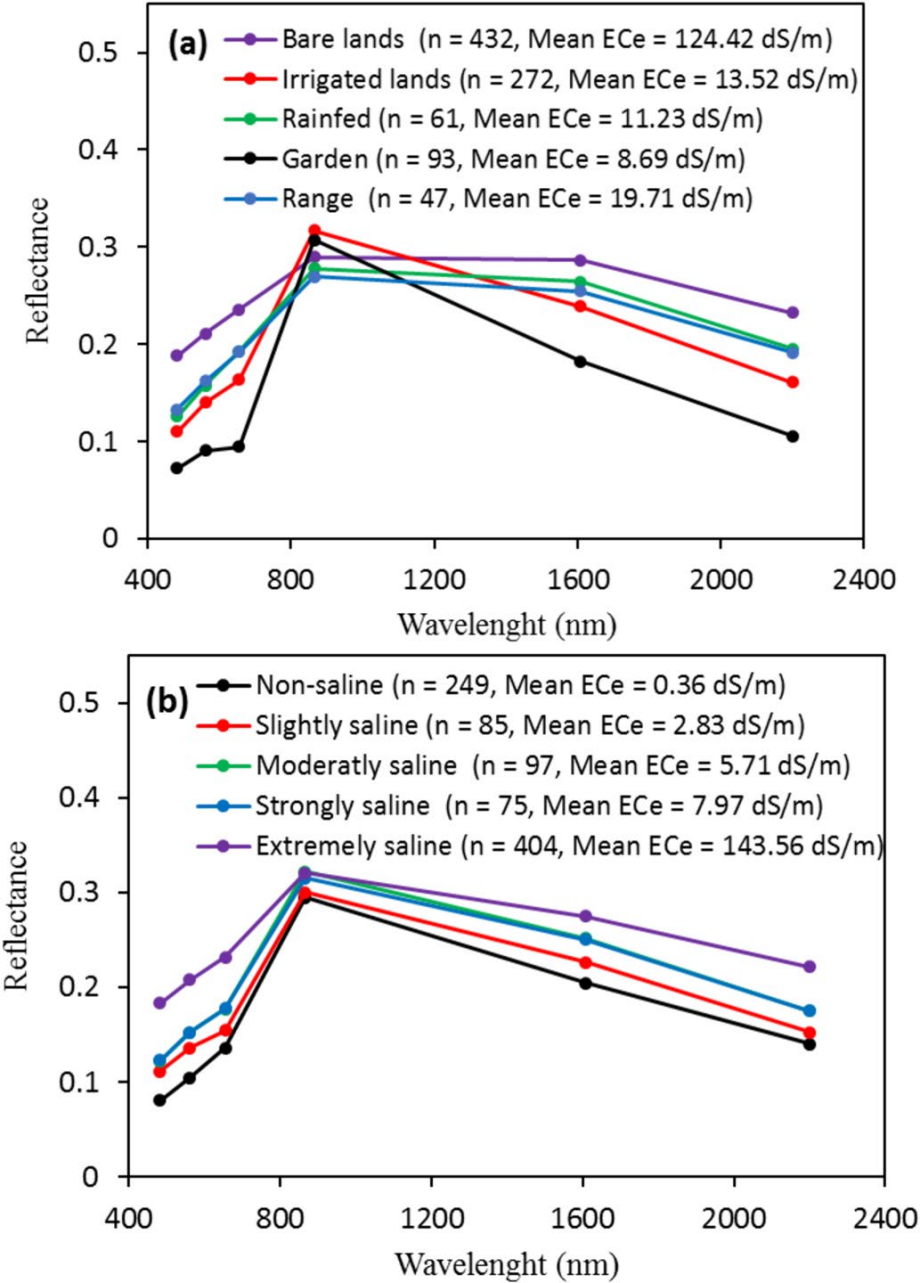
Reflectance spectra with different type of land uses (**a**) and salt-affected soils (**b**) in Lake Urmia region

Figure 5 illustrates the variations in mean spectral reflectance influenced by different land use types (Fig. 5a) and levels of soil salinity (Fig. 5b). Figure 5a demonstrates that bare lands and gardens exhibited the highest and lowest mean reflectance values in the Blue, Green, Red, NIR, SWIR1, and SWIR2 spectral bands. This discrepancy is likely related to the significantly higher mean soil salinity in bare lands, which averaged 124.42 dS m-1 (Fig. 5a). In contrast, irrigated farmlands and gardens displayed higher mean reflectance values than other land use categories in the NIR band (Fig. 5a). Figure 5b presents the mean spectral reflectance of salt-affected soils in the Lake Urmia region. Notably, an increase in soil salinity, ranging from non-saline conditions (with a mean of 0.36 dS m-1) to extremely saline conditions (with a mean of 143.56 dS m-1), resulted in an observable rise in reflectance. This finding aligns with similar observations reported in previous studies by Farifteh et al. (2006), El Harti et al. (2016), Wang et al. (2019), and Seifi et al. (2020), where reflectance values increased sharply in the VIS–NIR range with increasing soil salinity.

### Correlation analysis and develop a new spectral index

**Fig. 6:**
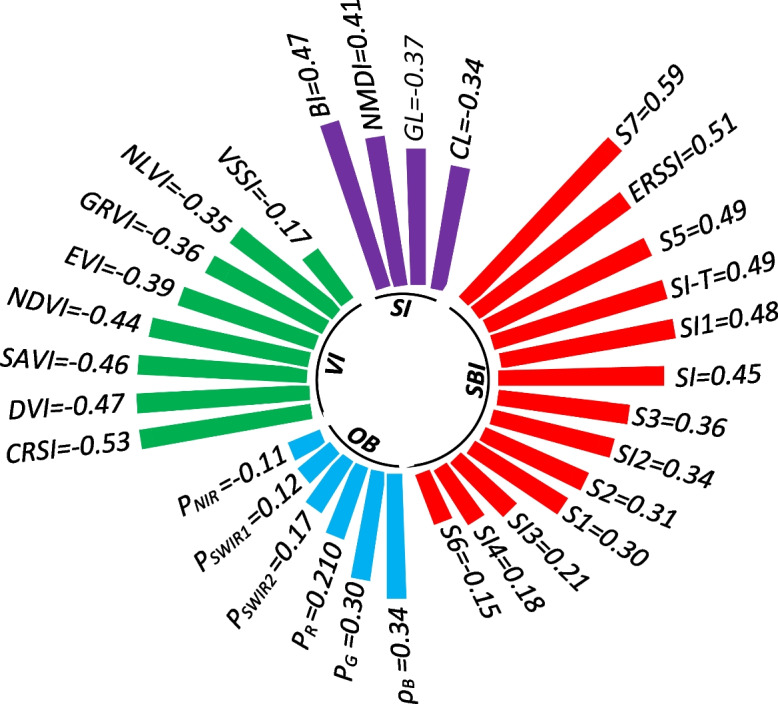
The correlation coefficient between EC_e_ data and spectral indices

The correlation coefficient analysis between ECe and the spectral indices from remotely sensed data is presented in Fig. 6. The correlation coefficients ranged from -0.11 to 0.34 for optical bands, -0.17 to -0.53 for vegetation-related indices, -0.37 to 0.47 for soil-related indices, and -0.15 to 0.51 for salinity-related indices (Fig. 6). However, it is evident that there is no single spectral index that can effectively predict soil salinity across all environmental conditions. Moreover, the study highlights those existing spectral indices developed in the literature exhibit limitations when applied to various land cover types and geographic environments (Chen et al., 2022; Wang et al., 2022). Therefore, there is an urgent need to develop a new spectral index that takes into account current agricultural practices, management strategies, and ecosystem health. Equation (6) was developed in this research as a soil salinity index for monitoring soil salinization.

6 $$S7=\left(\left(\rho_{NIR}\times\rho_R\right)-\left({{\rho }_{SWIR1}}^{0.5}-{{\rho }_{SWIR2}}^{0.5}\right)\right)/\left(\left({{\rho }_{B}}^{2}\times{{\rho }_{G}}^{2}\right)+\left({{\rho }_{SWIR1}}^{0.5}-{{\rho }_{SWIR2}}^{0.5}\right)\right)$$

The use of multi-year reflectance data (from 2016 to 2018) collected from various land uses (Fig. 5a) and different salt-affected soils (Fig. 5b) ensures the versatility of the developed index in diverse environmental scenarios. Figure 6 illustrates that the soil salinity index developed in this research (S7) exhibits the highest correlation coefficient (r = 0.59) with soil salinity.

In this study, the visible and near-infrared (VIS–NIR) as well as the short-wave infrared (SWIR) bands were employed to detect and monitor soil salinization when deriving the new spectral index. Most of the indices developed in the literature utilize the VIS–NIR bands for soil salinization detection, but some studies emphasize the significance of SWIR bands in predicting soil salinization (El Harti et al., 2016; Farifteh et al., 2006). This importance stems from the fact that SWIR bands are strongly associated with the salt's chemical composition and soil mineralogy (Wang et al., 2019). It is worth noting that the newly developed spectral index may have limitations in urban areas, where it may yield less accurate results.

### Develop machine learning models to predict soil salinity

**Fig. 7:**
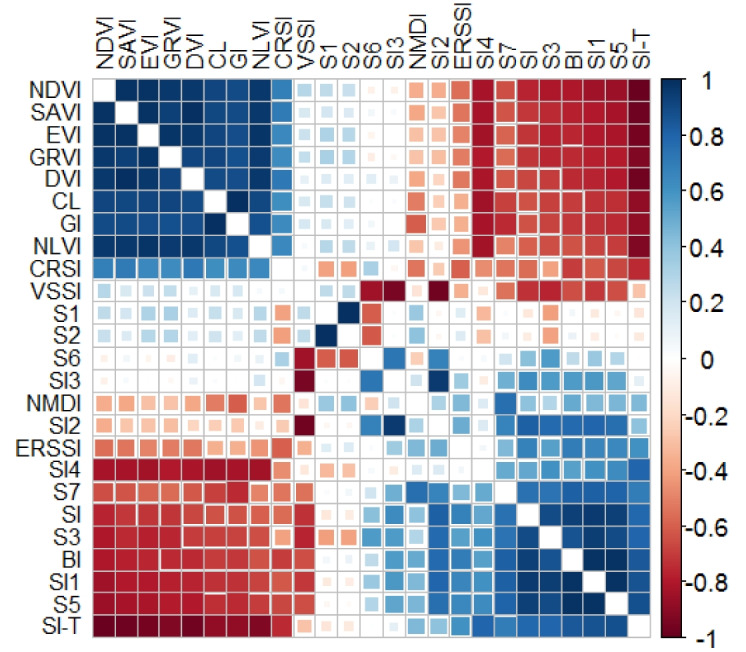
The correlation coefficient between different spectral indices

In this statistical study, the Pearson correlation test was used to determine the best combinations of input variables in different scenarios (Trifi et al., 2022). The correlation coefficients between the extracted spectral indices are depicted in Fig. 7. Further analysis of Figs. 6 and 7 revealed that the most promising combinations of spectral indices were as follows:

For scenario I, the best combination was S7.

In scenario II (direct spectral indices), the optimal combination included S7 and S2.

Scenario III (indirect spectral indices) showed that DVI and NMDI formed the most effective combination.

For scenario IV (direct plus indirect spectral indices), the ideal combination consisted of DVI, S7, and S2.

The results and performance of the developed regression and machine learning models in this study, using different scenarios of input variables (defined in Table 2), are presented in Table 5. In scenario I, the RF model performed the best, with ME = -4.432, R2 = 0.481, and RPIQ = 1.37. Introducing S2 to scenario I (scenario II) improved soil salinity prediction. Uncorrelated indices, including S7 and S2 (direct spectral indices), provided better results than the indirect spectral indices.

**Table 5:** The performance of various models in different scenarios to predict EC_e_ for test data set

Model	Formula	ME	R^2^	PRIQ	Mean VIF
LR					
Scenario I	$ECe=63.24+72.29S7$	-1.948	0.276	1.19	-
Scenario II	$ECe=119.38+74.62S7+391.67S2$	0.813	0.412	1.29	1.3
Scenario III	$ECe=-159.68-443.29DVI+417.43NMDI$	4.387	0.365	1.27	2.6
Scenario IV	$ECe=175.44-433.73DVI+466.91S2+45.91S7$	-1.832	0.471	1.35	4.1
SVM					
Scenario I	—	-6.342	0.468	1.33	-
Scenario II	—	-3.838	0.670	1.59	-
Scenario III	—	-11.918	0.629	1.48	-
Scenario IV	—	-9.678	0.751	1.78	-
RF					
Scenario I	—	-4.432	0.481	1.37	-
Scenario II	—	-4.302	0.659	1.54	-
Scenario III	—	-9.264	0.615	1.49	-
Scenario IV	—	-8.887	0.735	1.74	-

SVM was the best model for estimating ECe in both scenario II and scenario III, with ME = -3.838, R2 = 0.670, and RPIQ = 1.59 for scenario II and ME = -11.918, R2 = 0.629, and RPIQ = 1.48 for scenario III. Integrating salt-, soil-, and vegetation-based indices further enhanced the accuracy of ECe prediction (scenario IV), and SVM remained the best model (ME = -9.678, R2 = 0.751, and RPIQ = 1.78), closely followed by the RF model (ME = -8.887, R2 = 0.735, and RPIQ = 1.74).

Scatter plots of different models in scenario IV revealed consistent under-estimation of ECe, particularly by machine learning models like SVM and RF. The histogram of residuals for the train and test datasets in different models indicated that machine learning models had a well-distributed normal distribution of residuals. The SVM and RF methods showed very similar estimations for both the train and test datasets, emphasizing their high-performance capabilities.

Soil salinization is influenced by various environmental factors, and linear regression methods may not provide accurate predictions, especially in areas with high temporal and spatial variations in soil salinity. SVM, known for its flexibility and epsilon (ɛ)-insensitive loss function, demonstrated its effectiveness in predicting soil salinity, particularly in complex and dynamic environments like the study area. The findings of this study align with previous research highlighting the potential of SVM models in predicting soil properties (Were et al., 2015; Wu et al., 2018; Xu et al., 2018). However, the findings of this study are specific to the arid and semi-arid regions and may not be directly applicable to other regions with different environmental conditions and soil characteristics. Furthermore, high temporal and spatial variations in soil salinity can pose challenges for accurate predictions by developed models. For these reasons, future studies should focus on including more comprehensive datasets covering various environmental factors and longer temporal scales to improve model accuracy. Additionally, predictive models on different soil management practices and land use could enhance the generalizability of the models.

**Fig. 8:**
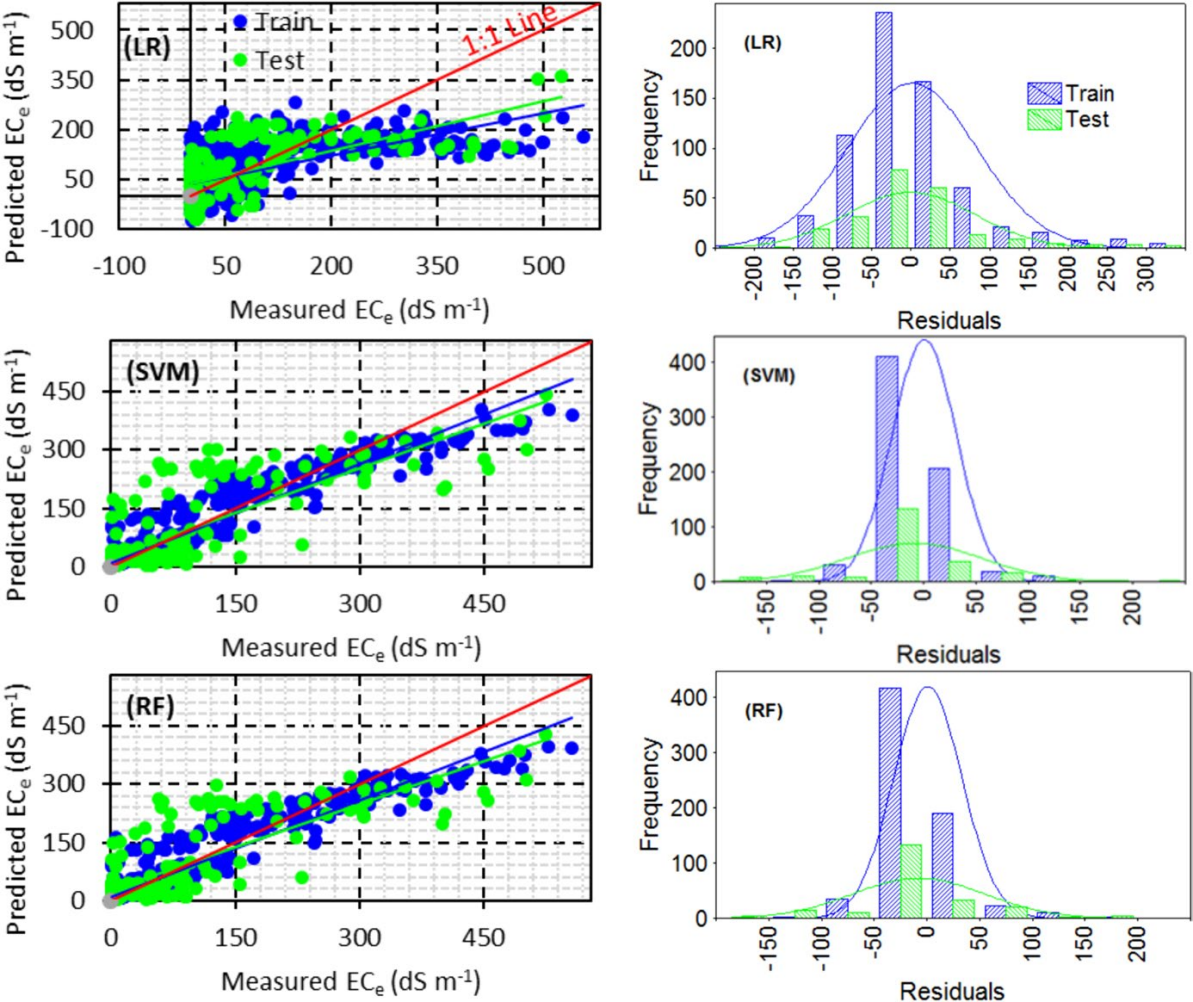
Scatter plots of the observed versus predicted EC_e_ and histogram of residuals for LR, SVM, and RF with the best strategy (i.e. scenario IV)

**Fig. 9:**
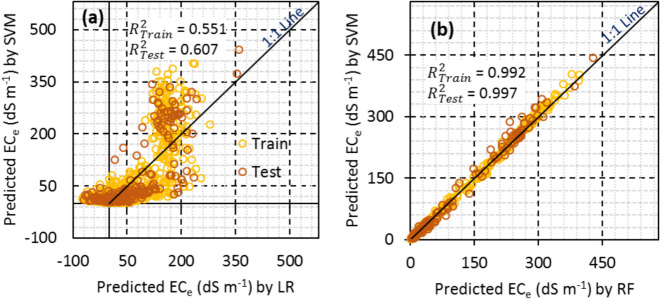
Comparison of predictions by SVM vs. LR (**a**) and SVM vs. RF (**b**) with the best strategy (i.e., scenario IV)

Overall, the study suggests that machine learning models like SVM and RF outperform linear regression in predicting soil salinity, especially in regions with dynamic and complex soil salinization patterns (Figs. 8 and 9).

### Predicted map of soil salinity in Lake Urmia region

**Fig. 10:**
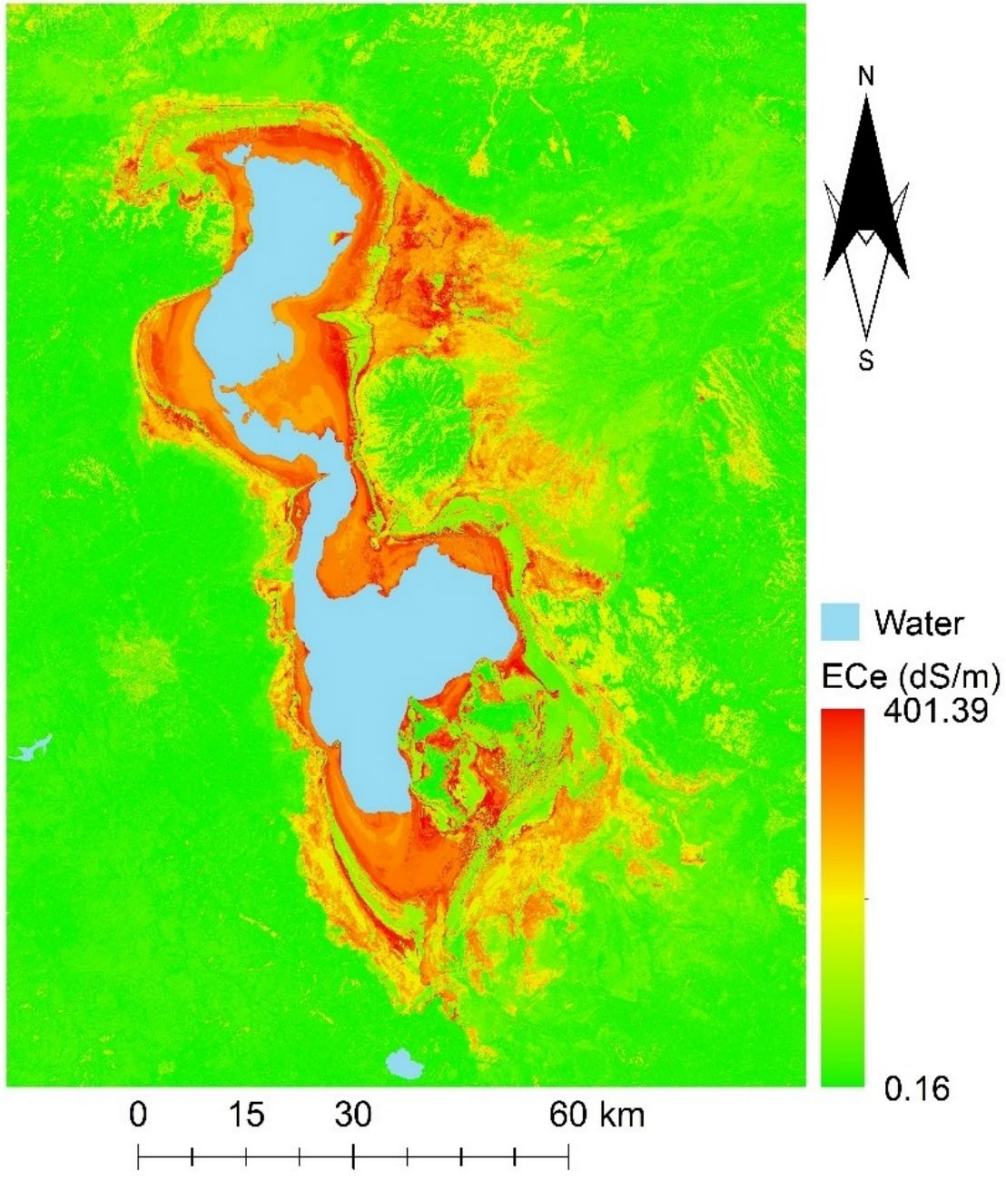
Soil salinity map by applying spectral indices (Scenario IV) extracted from Landsat images on September 2022 and by using the best model (i.e. SVM model)

In the current study, the best-performing model, which was the SVM model developed using scenario IV as input variables (comprising direct and indirect spectral indices), was utilized to create a soil salinity map (Fig. 10). The Lake Urmia region exhibited varying levels of salt-affected soil. Notably, there was a discernible decrease in soil salinity from the lake's vicinity toward the peripheries of the study area. However, it is worth noting that the distribution of soil salinity exhibited variations in different geographic directions (Fig. 10).

Several yellow-colored areas located farther from the center of study area, corresponded to urban regions, which appeared white, possibly due to roofing or construction materials (Fig. 10). This map provides a valuable representation of the spatial distribution of soil salinity across the Lake Urmia region, allowing for a comprehensive understanding of the salt-affected areas and their geographical patterns.

## Conclusion

This study aimed to assess the efficacy of remote sensing data in establishing associations between multi-year, field-measured soil salinity records and surface reflectance data, particularly spectral indices, collected from 2015 to 2018 in the Lake Urmia region, Iran. The developed spectral indices, categorized into salt-, soil-, and vegetation-based indices, exhibited varying correlations with soil salinity, spanning from -0.53 to 0.51. Nevertheless, a novel spectral index introduced in this study demonstrated an improved correlation coefficient of 0.59 with soil salinity.

The comparative analysis of linear regression (LR), support vector machine (SVM), and random forest (RF) models revealed that machine learning models, specifically SVM and RF, yielded superior predictive capabilities for soil salinity. In the context of scenario IV, which encompassed both direct and indirect spectral indices, the soil salinity prediction accuracies of the three models were ranked as follows: SVM > RF > LR. The SVM model, identified as the best-performing model, elucidated up to 75.1% of the variance in soil salinity.

The resultant soil salinity map depicted a noticeable decline in salinity levels from the proximity of the lake towards the outer perimeters of the study area. In summary, the novel spectral index exhibited enhanced proficiency in quantifying salt content, and the findings of this study hold valuable implications for monitoring and mapping soil salinity in arid and semi-arid regions, notably the Lake Urmia region. Subsequent research endeavors should prioritize the development of spectral indices tailored to diverse land-use scenarios.

## Data Availability

Data will be made available on request.
